# Bovine *in vitro* oocyte maturation and embryo culture in liquid marbles 3D culture system

**DOI:** 10.1371/journal.pone.0284809

**Published:** 2023-04-21

**Authors:** Giuliana de Avila Ferronato, Carolina Mônica dos Santos, Paola Maria da S. Rosa, Alessandra Bridi, Felipe Perecin, Flávio Vieira Meirelles, Juliano Rodrigues Sangalli, Juliano Coelho da Silveira

**Affiliations:** Department of Veterinary Medicine, Faculty of Animal Science and Food Engineering, University of São Paulo, Pirassununga, SP, Brazil; Sapienza University of Rome, ITALY

## Abstract

Despite the advances in *in vitro* embryo production (IVP) over the years, the technique still has limitations that need to be overcome. In cell cultures, it is already well established that three-dimensional culture techniques are more physiological and similar to the *in vivo* development. Liquid marble (LM) is a three-dimensional system based on the use of a hydrophobic substance to create *in vitro* microbioreactors. Thus, we hypothesized that the LM system improves bovine *in vitro* oocyte maturation and embryo culture. In experiment I, bovine cumulus-oocyte complexes (COCs) were placed for *in vitro* maturation for 22h in two different groups: control (conventional 2D culture) and LM (three-dimensional culture). We found that oocyte nuclear maturation was not altered by the LM system, however it was observed a decrease in expression of genes important in the oocyte maturation process in cumulus cells of LM group (*BCL2*, *EIF4E*, and *GAPDH*). In experiment II, the COCs were conventionally matured and fertilized, and for culture, they were divided into LM or control groups. There was a decrease in blastocyst rate and cell counting, a down-regulation of miR-615 expression, and an increase in the DNA global methylation and hydroxymethylation in embryos of LM group. Therefore, for the bovine *in vitro* embryo production, this specific three-dimensional system did not present the advantages that we expected, but demonstrated that the embryos changed their development and epigenetics according to the culture system.

## Introduction

*In vitro* embryo production (IVP) is a worldwide used technique as an alternative to numerous reproductive issues. In livestock, the IVP is responsible for a large part of bovine embryos produced by assisted reproductive techniques [1]. In humans, it is estimated that more than 8 million babies are born in the world through this tecnique [2]. Therefore, the IVP has a substancial importance for domestic species and human reproduction.

Despite its relevance, the IVP has some limitations that need to be overcome. The rate of oocytes *in vitro* matured that develop to the blastocyst stage is in average 30% [3], and these *in vitro* produced blastocysts have lower pregnancy rates as well as low resistance to cryopreservation [4]. *In vitro* produced embryos also show epigenetic differences when compared to *in vivo* produced embryos [5], which can be regulated by DNA methylation, post-translational modifications of histones and/or microRNAs (miRNAs), influencing during embryonic development and after birth [6].

Faced with the IVP limiting factors, the need arises to seek new perspectives that contribute positively to the *in vitro* culture of oocytes and embryos. The three-dimensional (3D) culture systems, are based on a cellular microenvironment similar to that found *in vivo*, which allows cells to interact with its surroundings on its three dimensions keeping the cells original structure during the *in vitro* culture [7]. Liquid Marbles (LM) is one type of 3D culture system, which is produced from highly hydrophobic particles that adhere to a drop of liquid, making a sphere that remain stable [8]. The droplet size can be variable, allowing for group or individual cultures, depending on the needs of each study. Also, a very low amount of culture medium volume can be used in addition to the ability that the drops allow good gas permeability at a low cost [9, 10].

The LM system has been widely used in cell cultures for different applications, such as to formation of organoids and spheroids [11–13]; to embryonic body formation, where it demonstrated excellent cell viability in 7-day cultures by live/dead assay and also in 48-hour cultures by MTT [14]. In studies focused on reproduction, it has already been used in sheep, where the nuclear maturation rates, cleavage, and blastocyst was similar between conventional maturation or using LM [15]. Moreover, in prepubertal ovine females where the nuclear maturation rates were similar, but there was an increase in the blastocysts rate from the oocytes matured in LM group [16]. Together these studies suggest that the LM system has no toxic effect on the cells and appears to improve *in vitro* embryo production.

Considering the importance of oocyte maturation for the IVP technique success and the importance of viable blastocysts production, we hypothesized that the LM system improve *in vitro* oocyte maturation and embryo culture in bovine. For that, this study was divided in two sets of experiments, which evaluated in an independent way the impacts of the LM culture on oocyte maturation and embryo developmental rates. Our findings demonstrate that the LM system does not alter the oocytes nuclear maturation rate, but may have a negative effect on cumulus cells from these oocytes and can also impair early embryonic development in bovine, contrary to our hypothesis.

## Materials and methods

All experimental procedures were approved by the College of Animal Science and Food Engineering, University of São Paulo (FZEA/ USP) Ethics Committee (protocol number: 5343150721) and did not involve human subjects. This work had two completely independent studies: experiment I: COCs *in vitro* maturation (IVM) and experiment II: *in vitro* embryo culture (IVC). Unspecified chemical brand is from Sigma‐Aldrich/Merck Chemical Company.

### Experimental design I—IVM

**Fig 1:**
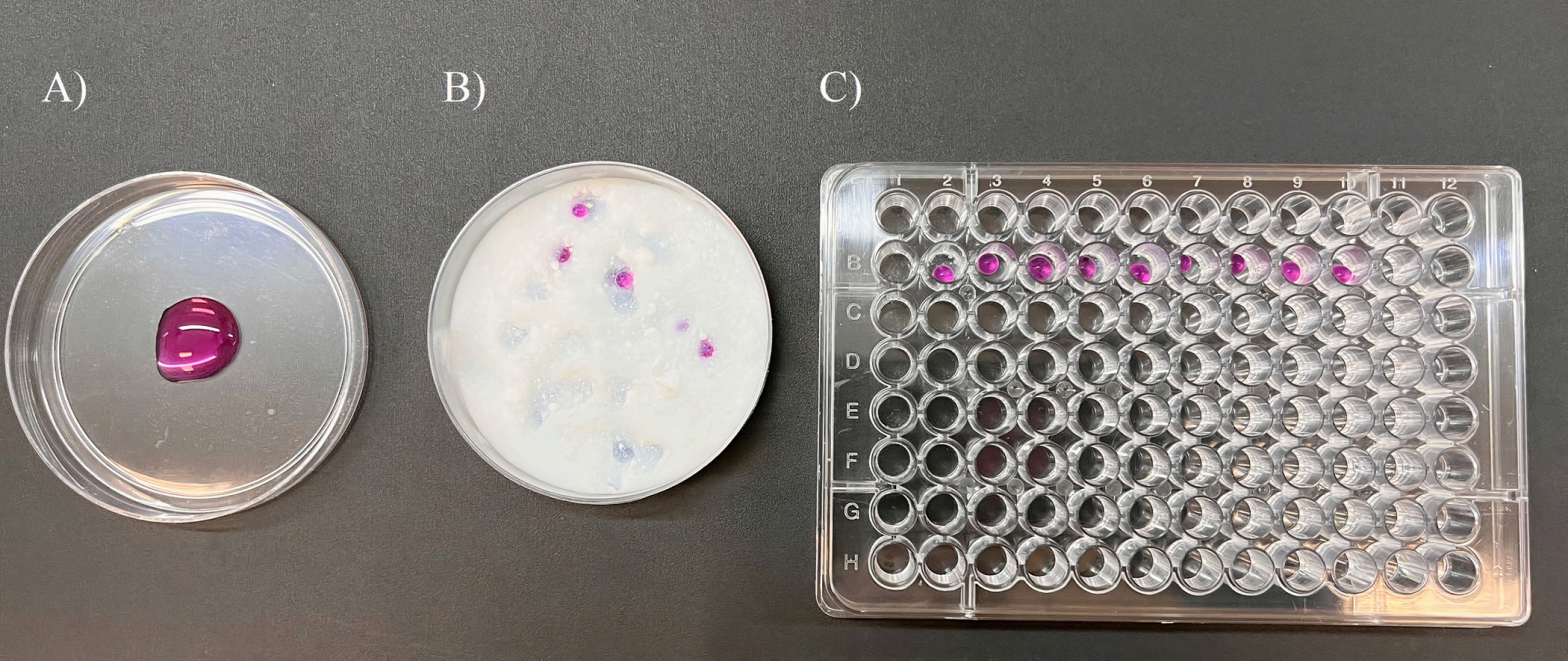
**Liquid marble (LM) culture system.** For both experiments (IVM or IVC), the LM system was performed in the same way, just changing the number of structures inside the microbioreactor. **A)** The structures to be cultured (COCs or presumptive zygotes) were all placed inside the same drop of culture medium in a petri dish. **B)** A total if 6 COCs (experiment I—IVM) or 3 presumptive zygotes (experiment II—IVC) were picked up in a 30 μl of culture medium and placed in contact with the CAB-O-SIL®TS-530 fumed silica powder to coat the drops. **C)** The drops were transferred using a 1000 μl pipette to a 96-well plate to be incubated at 38,5°C in 5%CO_2_.

For this experiment, COCs were selected according to their morphology (Grade I and II) and divided into two different groups for *in vitro* maturation. The experimental groups were: Control or LM. For the control group, the COCs were maturated in groups of 20 in a 100 μl drop of maturation medium (TCM 199 –GIBCO, buffered with 25 mM sodium bicarbonate, supplemented with 10% FBS, 0.2 mM sodium pyruvate, 50 μg/mL gentamicin sulfate, 0.5 μg/mL FSH—Folltropin and 5 U/mL hCG), submerged in 4 mL of mineral oil. In the LM group, COCs were maturated in groups of 6 COCs in drops of 30 μl of maturation medium (Fig 1), coated by CAB-O-SIL®TS-530 fumed silica (chemical name: Silanamine, 1,1,1-trimethyl- N-(trimethylsilyl)-silica hydrolysis products) and placed for culture in 96-well plates containing one drop in each well (Fig 1).

Seven biological replicates were performed for IVM rates (~30 COCs/group/replicate). From these, 5 biological replicates were used for gene expression of oocytes and cumulus cells. The oocytes were denuded together and snap frozen in pools containing 10 oocytes. The cumulus cells from these oocytes were briefly centrifuged and also snap frozen. Then the samples were stored at -80°C for further RNA isolation. For cumulus cell expansion, it was performed 3 replicates (60 COCs/group/replicate), and the pictures were taken before and after maturation.

### Experimental design II–IVC

IVM and IVF were performed conventionally for both groups as describe below. After 18 hours of the IVF, the presumptive zygotes were denuded through multiple pipetting, washed and divided into groups: control group and LM Group. The control group (n = 60/replicate) was conducted conventionally, on 4-well plates, with 500 μl SOFaaci culture medium (containing 2.5% FBS, 5 mg/ml BSA, 22 μg/ml sodium pyruvate and 50 μg/ml gentamicin) and 150 μl mineral oil per well, containing 30 presumptive zygotes per well. The LM group (n = 60/replicate) was conducted in microbioreactors (Fig 1), created with drops of 30 μl of SOFaaci culture medium, containing 3 presumptive zygotes per drop, coated with CAB-O-SIL®TS-530 fumed silic and placed for culture in 96-well plates containing one drop in each well (Fig 1). They were cultivated for 7 days at 38.5°C, 5% CO_2_, 5% O_2_ and 90% N_2_. The blastocysts were individually frozen by snap frozen and stored at -80°C for miR-615 expression or were fixed in 4% PFA and stored at 4°C for immunofluorescence analysis. Seven replicates were performed for miRNA expression and five replicates were performed for immunofluorescence (global DNA methylation and hydroxymethylation, blastocyst size, and cell counting).

### *In vitro* maturation

Cumulus-oocyte complexes (COCs) were aspirated from antral follicles (3–6 mm in diameter) from local slaughterhouse ovaries. The COCs were selected in TCM 199 medium modified with Hepes (supplemented with 0.001 g/mL of BSA, 0.2 mM of pyruvate and 50 μg/mL of gentamicin sulfate). COCs were selected according to their morphology (Grade I and II) and maturated for 22h, at 38.5°C, 5% CO_2_ in controlled humidity. For the control group, COCs were maturated in groups of 20 in a 100 μl drop of maturation medium (described above), submerged in 4 mL of mineral oil. For the LM system 6 COCs were placed in a 30 μl of media and used to creat the microbioreactor droplets. For experiment I, the oocytes were denuded to evaluate the nuclear maturation rate, then the oocytes and cumulus cells were collected, snap frozen in liquid nitrogen, and stored at -80°C for further analysis. For experiment II, after 22h of conventional IVM, the COCs proceeded to the *in vitro* fertilization (IVF) stage.

### Nuclear maturation rate

After 22h maturation, the oocytes from control group (n = 201) an LM group (n = 113) of 7 different replicates of experiment I (IVM) were denuded by multiple pipetting using TrypLE™ Express Enzyme (ThermoFisher). The completely denuded oocytes were washed in PBS 1% PVP and the first polar body extrusion was observed. The samples were stored in pools of 10 oocytes and the cumulus cells were centrifuged twice at 300 xg for 5 minutes and the supernatant was removed. Samples were first frozen by snap freezing in liquid nitrogen followed by storage at -80°C until RNA isolation.

### Cumulus cells expansion

This evaluation was performed in three replicates (n = 60 COCs/group/replicate) from experiment I. Images of the morphology of cumulus expansion were assessed using an inverted microscope (Nikon Eclipse TI) under 4× magnification of all the oocytes of each group before placing for maturation and after maturation. The cumulus cells expansion was measured using the Image J software circling the entire COC circumference.

### *In vitro* fertilization

For the experiment 2, after the IVM, the oocytes were washed and transferred to 100 μl drops of IVF-TALP (Tyrode-lactate stock) medium, supplemented with 6 mg/ml BSA, 5.5 IU/ml heparin, 40 μL/ml PHE (2 mM D-penicillamine, 1 mM hypotaurine, and 245 μM epinephrine), 22 μg/ml pyruvate and 50 μg/ml gentamicin, submerged in mineral oil. The semen straw (from the same bull and same batch) was previously processed in a Percoll gradient (45% and 90% concentration) to obtain a approximately final concentration of 25x10^6^ viable sperm/mL to be added to the drops where the oocytes were located and incubated at 38.5°C, 5% of CO_2_ in a controlled humidity.

### *In vitro* culture

The presumptive zygotes were denuded all together by multiple pipetting, into the same IVF drops they were placed before, and washed using the TCM 199 medium modified with Hepes described above. For each replicate, 120 presumptive zygotes were divided into two groups (60 COCs per group), previously described. In control group, they were divided into 2 wells on nunc plates, containing 30 presumptive zygotes per well. Each well contained 500 μl SOFaaci culture medium (containing 2.5% FBS, 5 mg/ml BSA, 22 μg/ml sodium pyruvate and 50 μg/ml gentamicin) and 150 μl mineral oil. The LM group was performed, using 3 presumptive zygotes per microbioreactor droplet using 30 μl of the SOFaaci medium (20 LM microbiorreactors per routine = 60 presumptive zygotes). After that, they were incubated for 7 days at 38.5°C, 6% CO_2_, 5% O_2_ (regulated by 90% N_2_) and controlled humidity. On day 7 of the culture (D7) the blastocysts were classified morphologically, according with Bó et al. [17] in early blastocyst, blastocyst, expanded blastocyst, and hatched blastocyst. We performed 11 replicates in total, and the IVC rate was evaluated in all of them. Seven replicates were used for gene expression analysis; thus, the blastocysts were individually frozen by snap frozen and stored at -80°C until RNA isolation. Moreover, the others 5 replicates were performed to obtain blastocysts for immunofluorescence analysis, thus the blastocysts were fixed in 4% PFA and stored at 4°C.

### RNA isolation

Total RNA was isolated from 5 pools of oocytes/group (n = 10 oocytes/pool). From these same oocytes pools, the cumulus cells samples were recovered (5 samples/group) and total RNA was isolated. For blastocysts, the RNA was isolated from 3 pools containing 5 blastocysts in each pool (2 blastocysts and 3 expanded blastocyst), from 7 biological replicate. Total RNA from all samples was isolated with QIAzol Lysis Reagent (Qiagen), following the manufacturer’s protocol, in combination with 1.33 μl GlycoBlue co-precipitant (Thermo Fisher Scientific). The quality and concentration of the RNA was evaluated in NanoDrop One (Thermo Fisher Scientific) and the total RNA was treated with DNAse I (Invitrogen; Carlsbad, CA) to avoid DNA contamination. One pool of RNA isolated from cumulus cells of the LM group was excluded from the analysis because of the result obtained in the nanodrop.

### cDNA synthesis and RT-qPCR for gene expression analysis

The cDNA was synthesized using the commercial iScript Synthesis kit (BIORAD®, Hercules, USA) following the manufacturer’s instruction. Real-time PCR was conducted with 7.5 ng per well analysed using the GoTaq qPCR Master Mix (Promega®, USA). In the experiment I (IVM), we evaluated the following genes in cumulus cells: *EIF4B*; *EIF4E*; *BAX*; *BCL2*; *CDK6*; *HAS2*; *GAPDH* and; *FOXO3a*. In oocytes: *GDF9*; *BMP15*; *PI3K*; *PTEN*; *FOXO3a*; *BAX* and; *BCL2*. The *ACTB* and *PPIA* genes were used as endogenous controls. Primers sequences are listed on Table 1. Statistics was performed with the normalized data and the transformed data (2^−ΔCt^) were used in the graphs.

**Table 1:** Bovine primer sequences used in the RT-qPCR amplification.

Gene symbol	Primer sequences (5’-3’)	Accession number	Reference
*ACTB*	**F:** CAGCAGATGTGGATCAGCAAGC	NM_173979.3	[18]
	**R:** AACGCAGCTAACAGTCCGCC		
*BAX*	**F:** CCCGAGTTGATCAGGACCAT	NM_173894.1	Andrad et al. [19]
	**R:** CACTCCAGCCACAAAGATGG		
*BCL2*	**F:** CTTTGTGGAGCTGTATGGC	NM_001166486.1	Andrade et al. [19]
	**R:** CCAGATAGGCACCCAGGG		
*BMP15*	**F:** GCCTCGGATCTTAGGGCATC	NM_001031752.1	Designed by authors
	**R:** TATGTGCCAGGAGCCTCTGA		
*CDK6*	**F:** CTCCGAGGCCTGGACTTTCT	NM_001192301.1	Andrade et al. [19]
	**R:** TAGATGCGAGCAAGGCCGAA		
*EIF4B*	**F:** ACGACTCCAGATCTGCACCTG	NM_001035028.2	Andrade et al. [19]
	**R:** TCTTCACCGTCAATGGCGAGA		
*EIF4E*	**F:** TTAATGCCTGGCTGTGACTAC	NM_174310.3	Andrade et al. [19]
	**R:** ACGATCGAGGTCACTTCGTCT		
*FOXO3*	**F:** GCAGGGAGCGCGATATTG	NM_001206083.1	Andrade et al. [19]
	**R:** CGGGCACCATGAATCTGAA		
*GAPDH*	**F:** GCCATCAATGACCCCTTCAT	NM_001034034.2	Designed by authors
	**R:** TGCCGTGGGTGGAATCA		
*GDF9*	**F:** CAGCCAGATGACAGAGCTTTGAG	NM_174681.2	Designed by authors
	**R:** CACTGATGGAAGGGTTCCTGCT		
*HAS2*	**F:** CCTAAACATTTGAGACTCCCCC	NM_174079.3	Designed by authors
	**R:** CACAATGCATCTTGTTCAGCTC		
*PI3K*	**F:** TCAAACGTGAAGGCAACGAG	NM_174575.1	Designed by authors
	**R:** CGCCTGCTTCTTCAAGTCCT		
*PPIA*	**F:** GGTCCTGGCATCTTGTCCAT	NM-001077866.1	[20]
	**R:** TGCCATCCAACCACTCAGTCT		
*PTEN*	**F:** GCCACAAAGTGCCTCGTTTACC	XM_613125.6	[20]
	**R:** AGAAGGCAACTCTGCCAAAC		
bta-miR-541	**F:** TGGTGGGCACAGAATCCGGCCT	NR_031188.1	[21]

### cDNA synthesis and RT-qPCR for miRNA expression

The cDNA was synthesized from 100 ng of total RNA with the miScript II RT kit (Qiagen), following the manufacturer’s instructions, using the miScript HiSpec Buffer for selection of mature miRNAs. For the bta-miR-615 expression, the RT-qPCR was performed using Power SYBR Green PCR Master Mix (Applied Biosystems), miScript Universal Primer (Qiagen), 0,05 pg cDNA per miRNA evaluated, and specific forward primer (10μM; the sequence is listed on Table 1). The device used was the QuantStudio 6 Flex (Applied Biosystems), under the following conditions: 95°C for 15min, followed by 45 cycles of 15 s at 94°C, 30 s at 55°C, and 30 s at 70°C and finally the melting curve. Ct values were normalized from bta-miR-99b to calculate the relative expression. The data were transformed through 2^-ΔCt^ to obtain the graphs.

### Global DNA methylation and hydroxymethylation

On day 7, the blastocysts were collected, fixed in 4% PFA and stored at 4°C for further analysis. Changes in global DNA methylation and hydroxymethylation were evaluated by immunofluorescence to detect 5-methylcytosine (5-mC) and 5-hydroxymethylcytosine (5-hmC). The embryos were incubated with PBS containing 1% Triton X-100 for 30 min, incubated in 4N HCl for 10 min, neutralized in 100 mM Tris-HCl (pH 8.5) for 20 min. Next, the embryos were placed in PBS with 3% BSA and 0.3 M Glycine for 1 hour. Finally, the embryos were incubated with mouse-specific anti-5-mC (Abcam; ab10805) and rabbit-anti-5-hmC (Abcam; ab214728, 1:500) antibodies, diluted at a concentration of 1:1000 in PBS overnight at 4°C. After 6 washes, the embryos were incubated with goat/anti-Mouse IgG-AlexaFluor 488 (Life Tech, cat. #: A-11029), and goat/anti-rabbit IgG-AlexaFluor 594 (Life Tech, cat. #: A-11012) secondary antibody for 1 h. In total, 7 blastocysts per group were analyzed by Leica SP5 confocal microscopy. All images were captured under the same parameters, performing sequential acquisitions. For visualization of methylation excitation and emission, it was set to 488 nm and 516 nm, respectively. For hydroxymethylation excitation and emission, it was set at 543 nm and 574 nm, respectively. Confocal images of the blastocysts were captured under a 40x objective, with 3 slices at different points for each blastocyst. The analyzes were performed using ImageJ software, measuring the fluorescence of all blastomeres present in each image, discounting the background fluorescence.

### Statistical analysis

Data are displayed in text and graphs as mean ± standard error of the mean. Statistical analyzes were performed using the GraphPad Prism 7 software. Outliers were identified and excluded, and the normality test was performed in the clean data by Shapiro-Wilk test. Frequency analysis (maturation and blastocyst rates) were performed using chi-square. For normal distribution data Student’s t test was performed (miR and mRNA expression, blastocyst size and cell counting). For non-normal data Wilcoxon test was used (cumulus cells expansion, global DNA methylation, and hydroxymethylation). A statistical difference was considered when P<0.05.

## Results

### Experiment 1: *In vitro* maturation of cumulus-oocyte-complexes

#### The effects of liquid marble culture applied to cumulus-oocyte complexes during *in vitro* maturation

**Fig 2:**
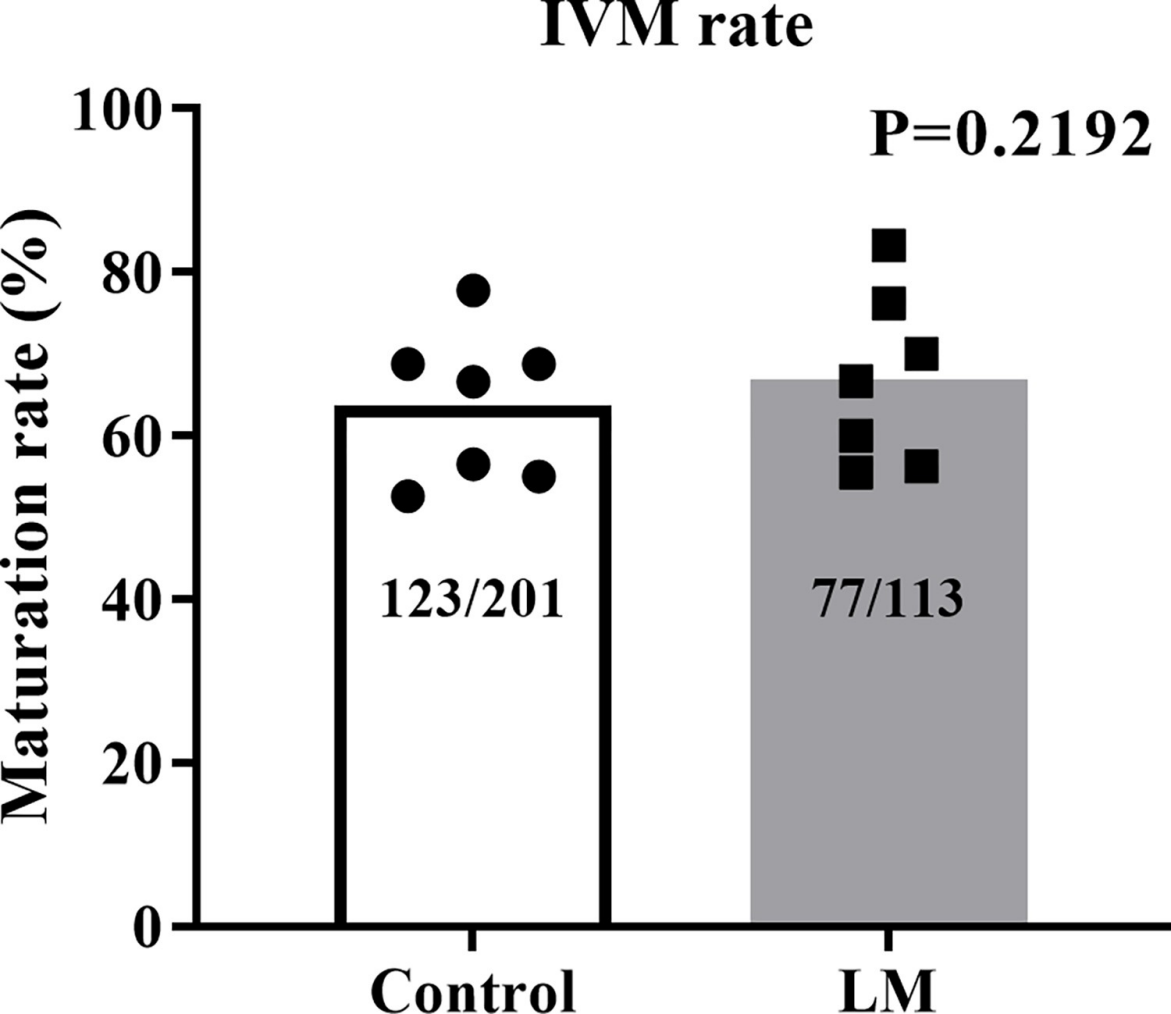
*In vitro* maturation rates. Nuclear maturation rate of oocytes cultured in conventional (control; 61.19%, 7 replicates) or in LM (68.14%, 7 replicates) systems. Within the bars there are mature/total oocytes numbers. Analysis were performed by Chi-square test with a statistical, no statistical difference was found between the groups (P>0.05).

**Fig 3:**
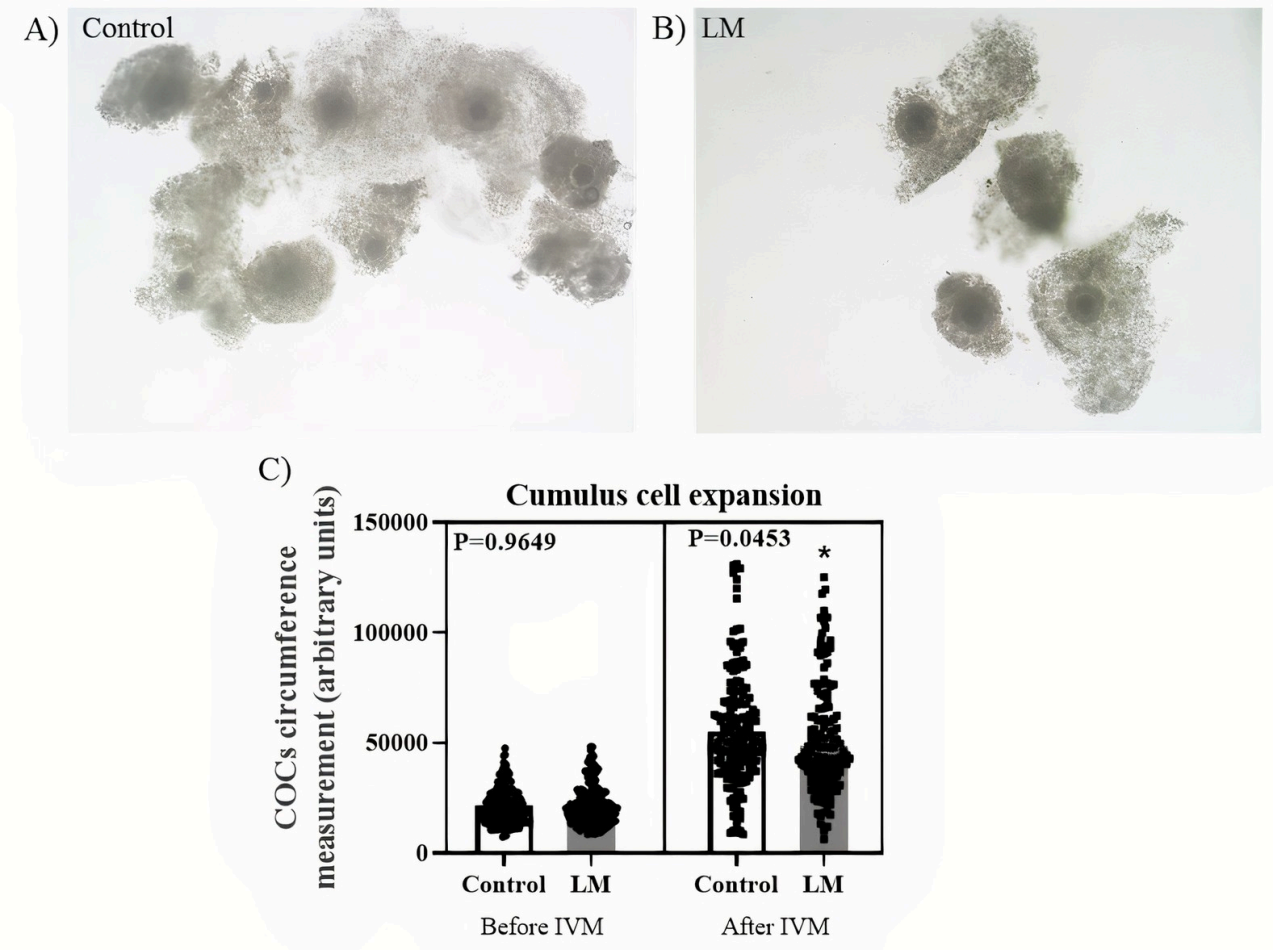
Cumulus cells expansion analysis. **A)** Cumulus-oocyte-complexes after IMV in control and **B)** LM systems. **C)** Cumulus cells expansion before IVM in control (n = 180) or LM system (n = 180), and after IVM in control (n = 172) or LM (n = 154) systems. Circumference of COCs were measured using ImageJ software. Analysis were performed using Wilcoxon test and asterisk represent statistical difference, considered when P<0.05.

To observe the effects of the LM system during IVM, COCs were submitted to IVM for 22 hours using the LM system and evaluated in comparison to a control group cultured in the conventional system. After IVM, the oocytes from both groups were denuded and the nuclear maturation rate was evaluated by the first polar body extrusion. The nuclear maturation rate did not differ (P = 0.2192) between the control (61.19%; n = 123/201) and LM systems (68.14%; n = 77/113) groups (Fig 2). Although, the cumulus cells showed a lower expansion in the LM group after maturation (P = 0.0453; Fig 3). Thus, these results demonstrate that the culture system by LM may affect the oocyte maturation supported by a lower cumulus cells expansion.

Target transcripts in cumulus cells and oocytes matured in different systems

**Fig 4:**
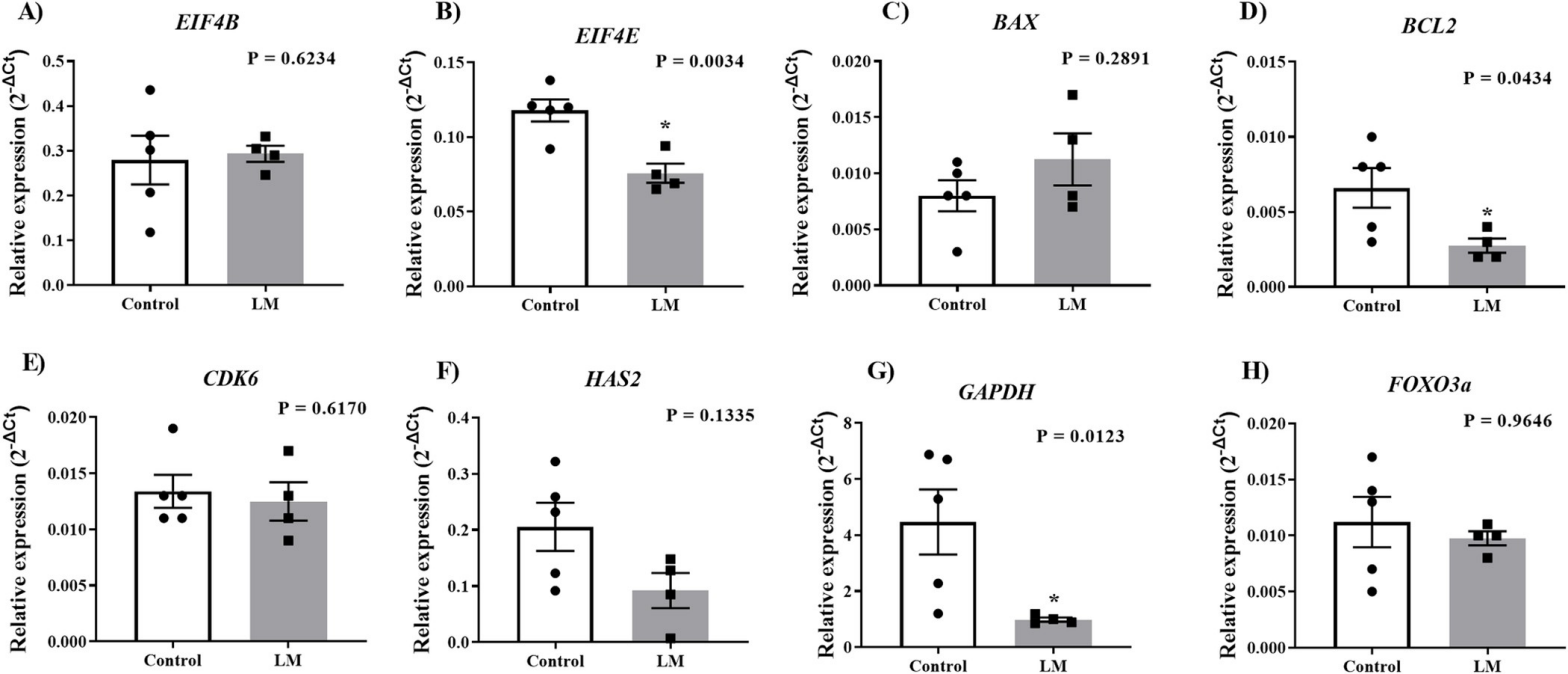
Target transcripts analysis in cumullus cells. Relative expression of **A)** *EIF4B;* **B)** *EIF4E*; **C)** *BAX;* **D)** *BCL2;* **E)** *CDK6;* **F)** *HAS2;* **G)** *GAPDH* and; **H)** *FOXO3a* genes in cumulus cells from COCs matured for 22h in conventional or LM systems (5 biological replicate). Transcripts levels analysis were compared using Student’s t test and asterisk means statistical difference, considered when P<0.05. Standard bars represents the SEM.

**Fig 5:**
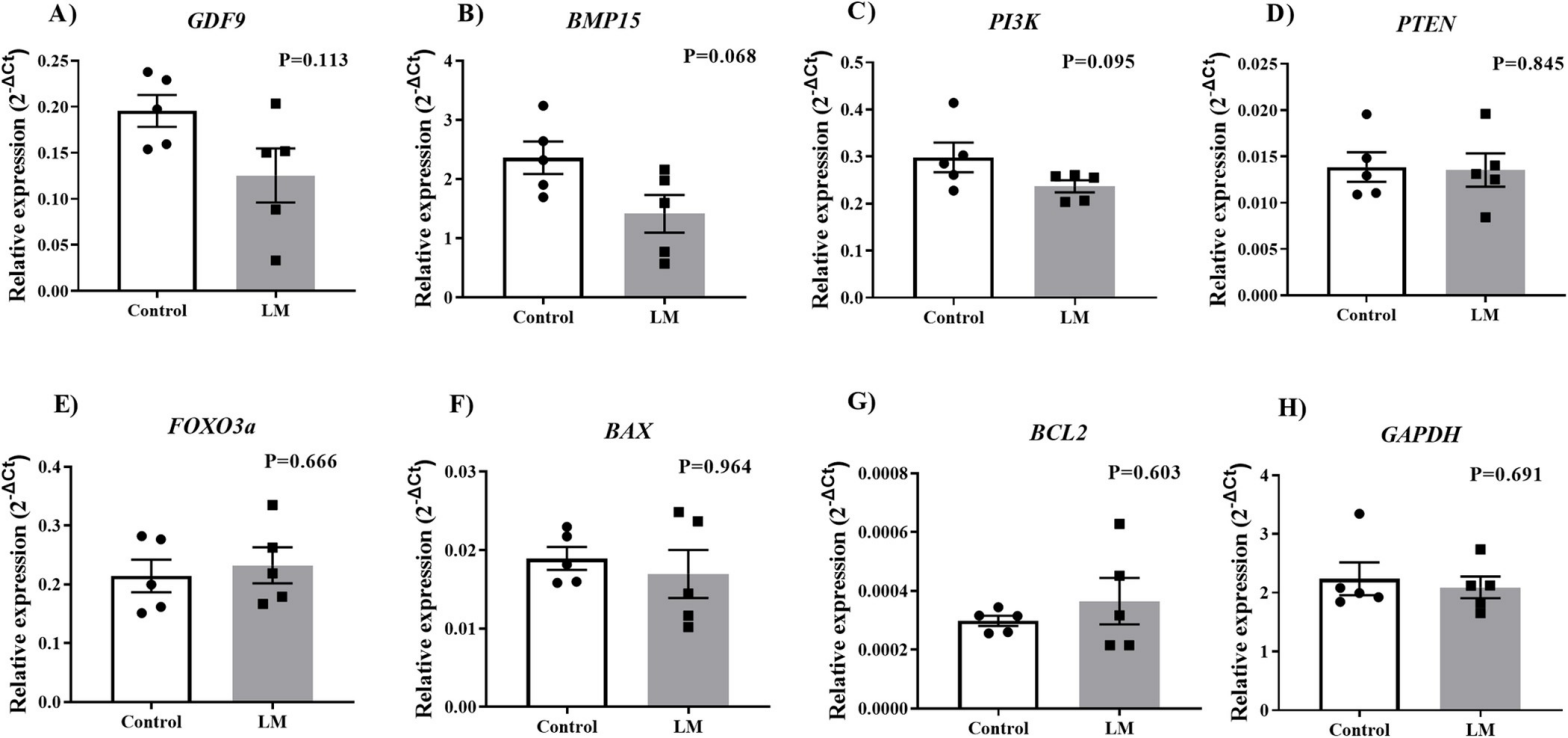
Target transcript analysis in oocytes. Relative expression of **A)** *GDF9;* **B)** *BMP15*; **C)** *PI3K;* **D)** *PTEN;* **E)** *FOXO3a;* **F)** *BAX;* **G)** *BCL2* and; **H)** *GAPDH* genes in oocytes matured for 22h conventional or LM systems (n = 10 oocytes/replicate, 5 biological replicates). Statistics was performed using Student’s t test, no statistical difference was found between the groups (P>0.05). Standard bars represents the SEM.

To evaluate the effects of the LM microbioreactors during IVM, we investigated the expression of target genes in cumulus cells and denuded oocytes isolated from the COCs maturated in control or LM systems. In cumulus cells (Fig 4A–4H), we observed decreased levels of three target genes (P<0.05), which had their expression reduced in LM when compared to the control group: *EIF4E*, *BCL2* and *GAPDH* (Fig 4B, 4D and 4G). Interrestingly, in denuded oocytes (Fig 5A–5H), we did not observed significant difference in the levels of the evaluated transcripts (P>0.05). In summary, the LM system may negatively modulate target transcripts in cumulus cells.

### Experiment 2: *In vitro* culture of presumptive zygotes

#### Blastocysts cultured inside LM microbioreactors had their development negatively affected

**Fig 6:**
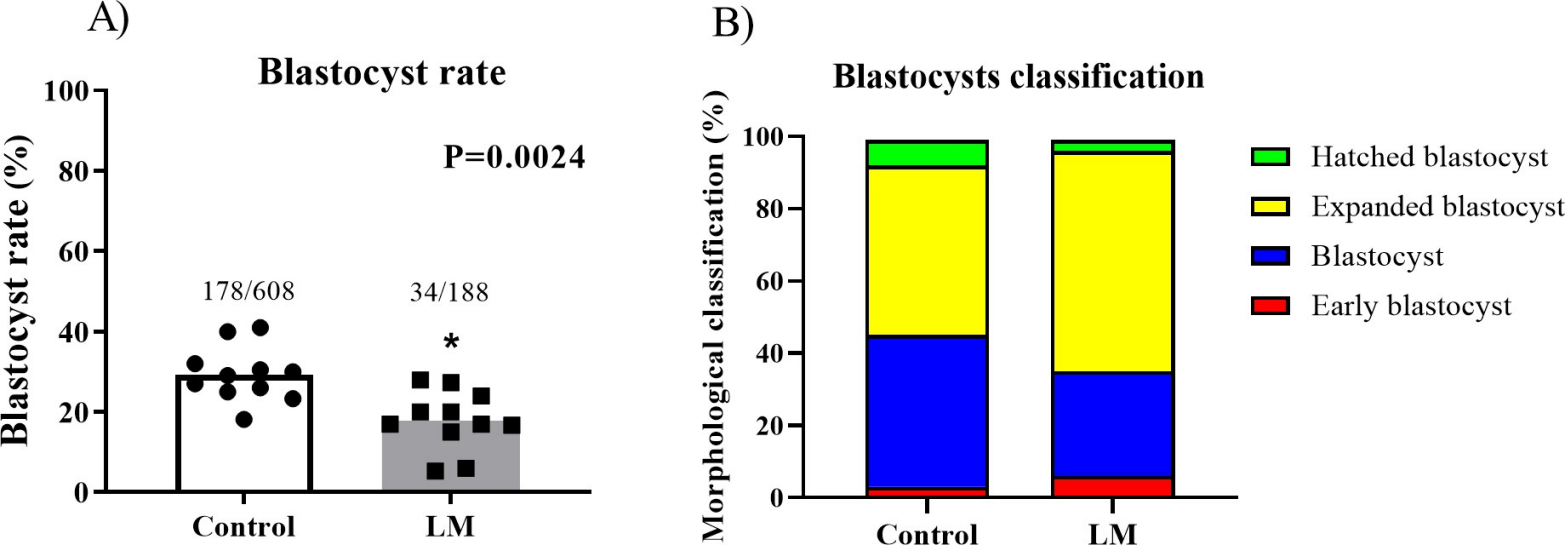
*In vitro* culture of presumptive zygotes. **A)** Total blastocysts rate obtained from the presumptive zygotes cultured for 7 days in conventional (control; 29.28%, 11 biological replicates) or in LM system (18.09%, 11 biological replicates). Statistics performed by chi square test, asterisk means statistical difference (P<0.05). **B)** Blastocysts morphological classification Bó and Mapletoft (17), from the 11 different routines. Standard bars represents the SEM.

**Fig 7:**
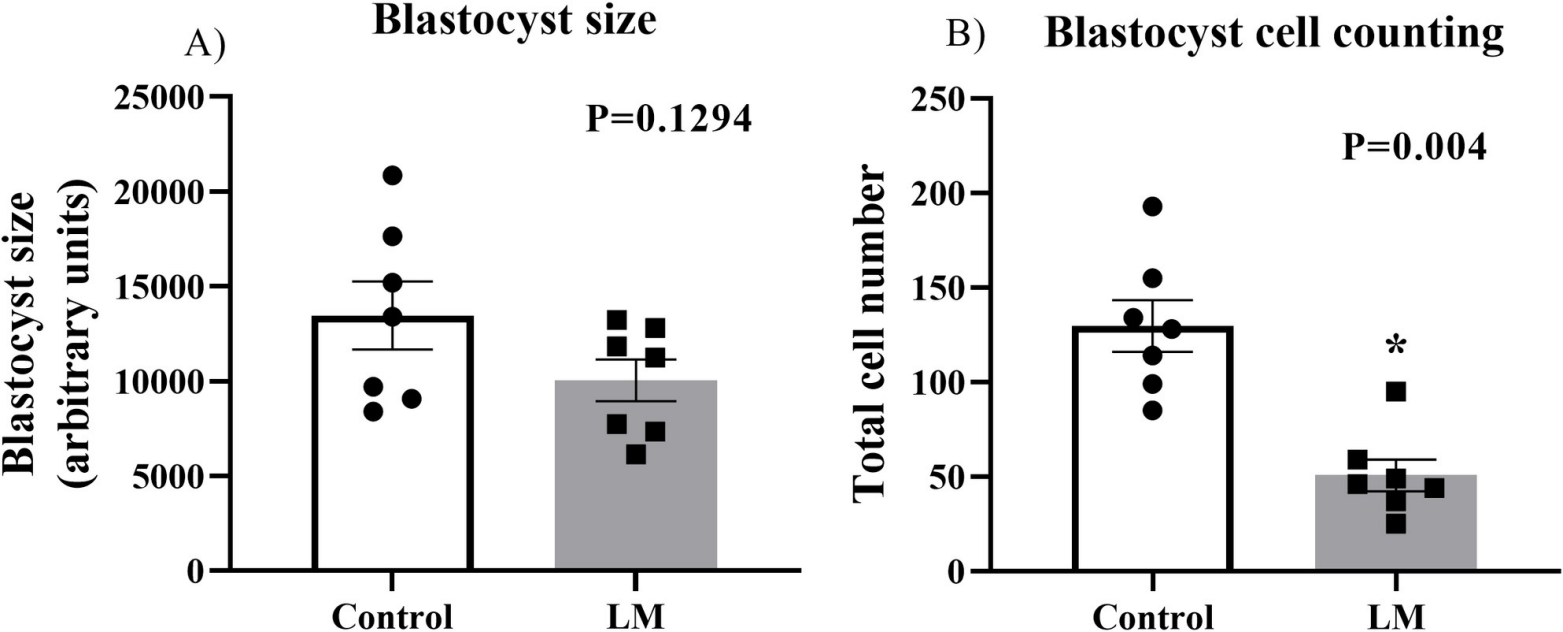
Blastocysts analysis. **A)** Blastocysts size (n = 7); and **B)** Total cell counting of day 7 blastocysts (n = 7) from control or LM group. Statistics performed by Student’s t test, asterisk means statistical difference (P<0.05). Standard bars represents the SEM.

In view of the results obtained in the IVM, we investigated how this LM system would affect the early embryo development. For that, we carried out an experiment to evaluate the use of LM only in the *in vitro* embryo culture (IVC). Thus, the COCs were matured and fertilized conventionally and the presumptive zygotes were cultured in LM or control systems until day 7 (D7). First, we aimed to evaluate the development of these blastocysts. We observed a lower blastocyst rate (P = 0.0030; Fig 6A) in the LM (18.09%; n = 34/188) compared to the control group (29.28%; n = 178/608). Although the overall blastocyst rate was different, the morphological classification of these blastocysts (early blastocyst, blastocyst, expanded blastocyst, and hatched blastocyst) appears to be homogeneous between the groups (Fig 6B), since the LM group, despite having more expanded blastocysts, also has a higher rate of initial blastocysts and a lower rate of hatched blastocysts. Additionally, the blastocysts did not present different sizes between groups (P = 0.1294; Fig 7A). However, the total number of cells of the blastocysts were significantly decreased in the LM group compared to the control (P = 0.004; Fig 7B). Therefore, these results suggest that the LM system negatively impacts blastocyst development.

#### Bta-miR-615 was down-regulated in blastocysts cultured in liquid marbles system

**Fig 8:**
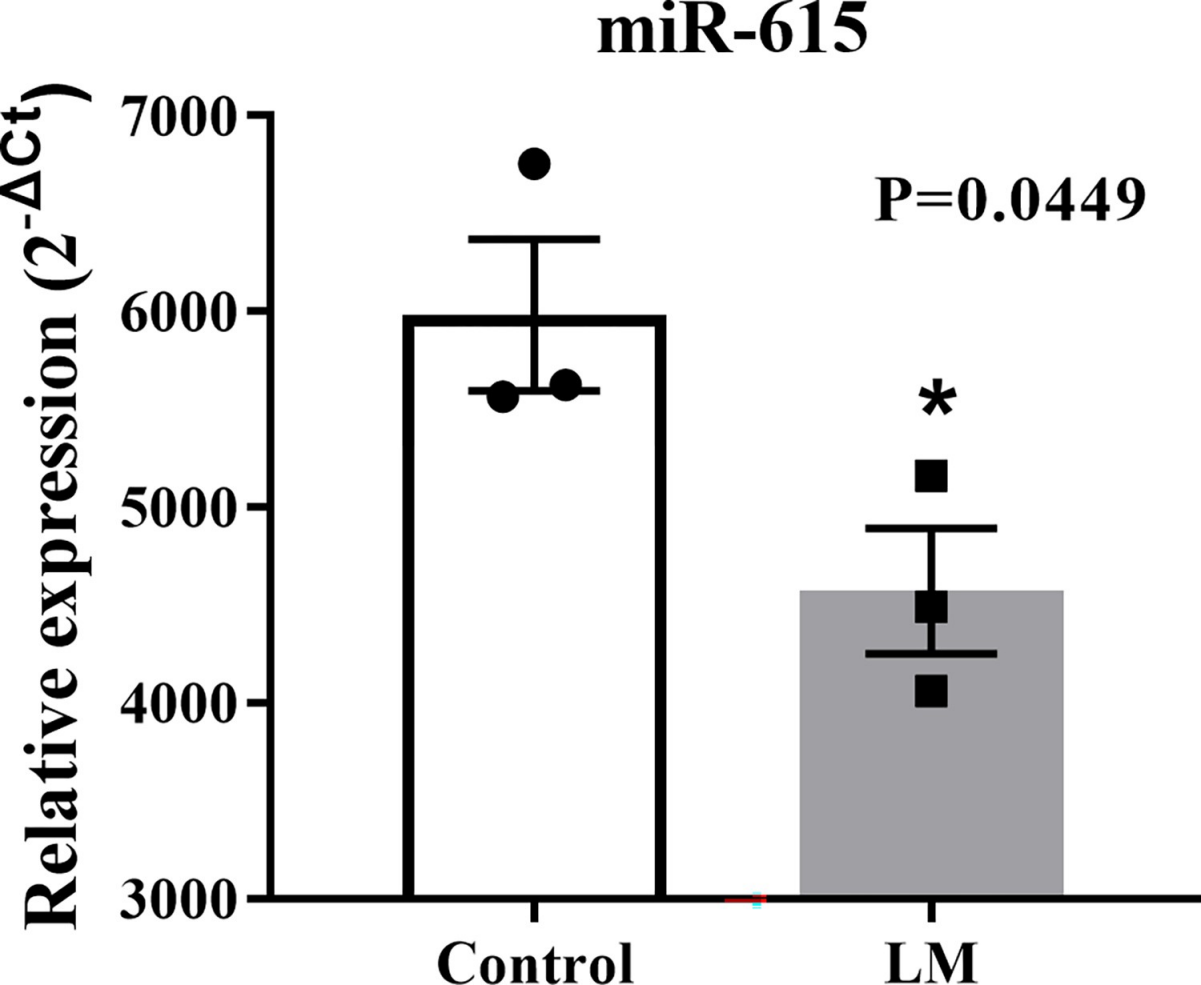
Bta-miR-615 analysis in blastocysts. Bta-miR-615 relative expression miRNA in blastocysts cultured conventionally (control) or in LM system. Statistics was performed using Student’s t test. Asterisk means statistical difference (P<0.05). Standard bars represents the SEM.

In order to evaluate the molecular effects of these two different culture systems at the level of post-trancriptional regulators, we investigated bta-miR-615 relative expression as a biomarker for embryo quality and development [22]. Bta-miR-615 was differently expressed (P = 0.0449), its expression was decreased in the LM group in comparison to the control group (Fig 8). Thus, indicating that LM affects embryo quality at the levels of post-trascriptional regulator as miRNAs.

#### Liquid marbles culture system increased blastocyst global DNA methylation and hydroxymethylation

**Fig 9:**
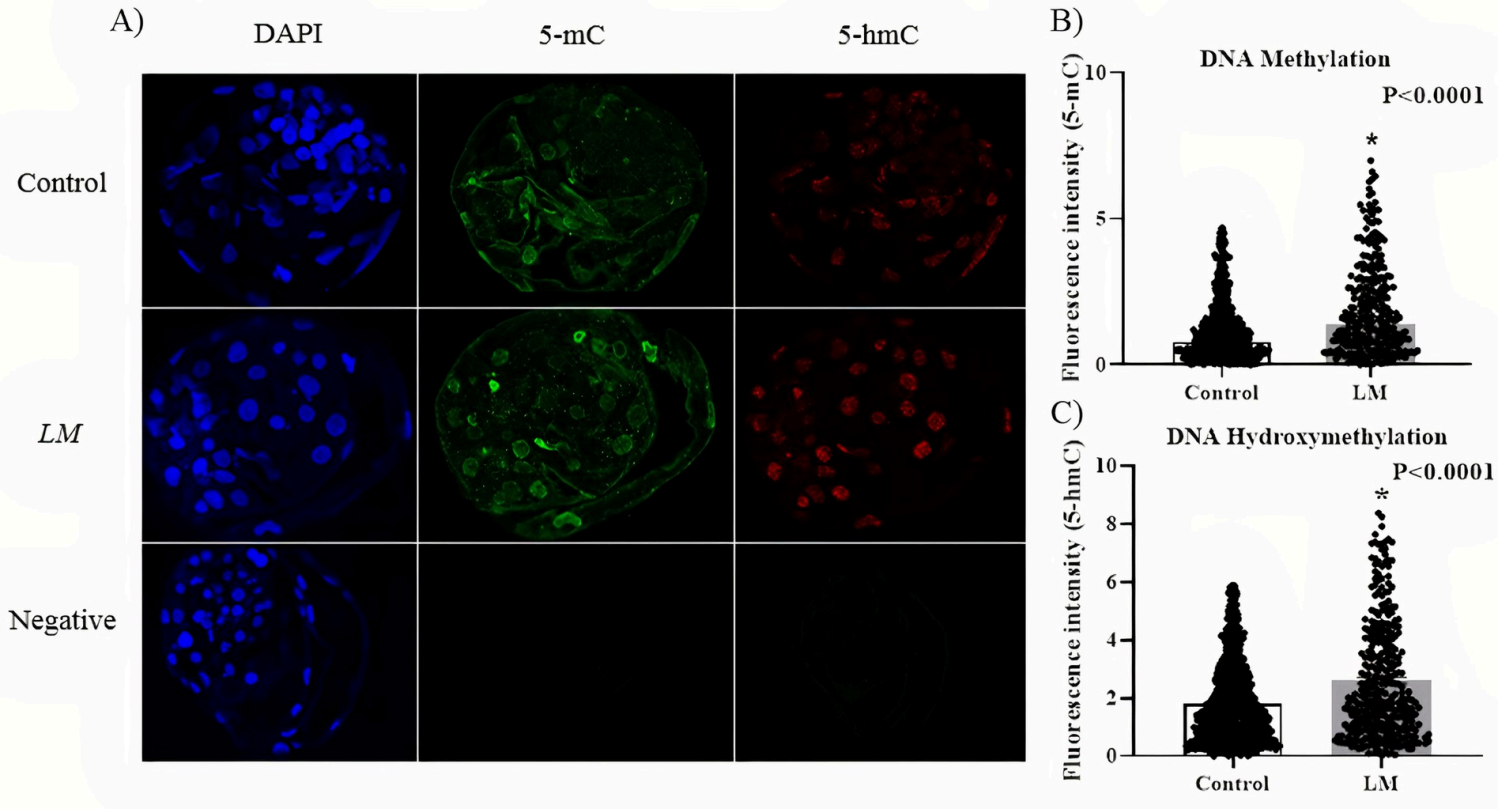
Global levels of DNA methylation and hydroxymethylation in blastocysts cultured in different culture systems. **A)** Confocal images of blastocysts submitted to different culture systems, conventional (control) or Liquid marble (LM), also a negative control was added to the experiment, without primary antibody labeling (40x objective). **B)** Global DNA methylation levels in nuclei (n = 909) of D7 blastocysts (n = 7) cultured in conventional system and in nuclei (n = 354) of D7 blastocysts (n = 7) cultured in LM systems. **C)** Global DNA hydroxymethylation levels in D7 blastocysts. Data are presented as mean ± SEM. Analysis were performed using Wilcoxon test. Asterisk represents statistical difference between groups (P<0.05).

To investigate the relation between culture environment (Control or LM) and epigenetic status we evaluated the levels of global DNA methylation in blastocysts using an antibody against 5-methylcytosine (5mC) and global DNA hydroxymethylation using an antibody against 5-hydroxymethylcytosine (5-hmC) in bovine blastocysts (Fig 9A). The results demonstrated that embryos cultured in the LM system had a higher levels of both DNA methylation (P<0.0001; Fig 9B) and hydroxymethylation (P<0.0001; Fig 9C) than those cultured in the conventional system. Thus, these results indicate that a series of genes may be differently regulated in the experimental groups, supported by the increase of both methylation and hydroxymethylation in the LM system.

## Discussion

The oocyte quality directly influences the embryo development. Among the different steps involved in oocyte quality, the maturation is the most important process, allowing this cell acquire the necessary competence to be fertilized and develop into blastocyst. Although *in vitro* nuclear maturation rates are high in the literature [23, 24], most of these oocytes do not develop into a blastocyst *in vitro*. Thus, the *in vitro* culture plays a pivotal role determining the oocyte and embryo quality. Based on that, in the present study we evaluated a different type of *in vitro* culture system, based on liquid marbles (LM). In order to investigate the effects of LM system we performed experiments evaluating the IVM and IVC independently. For our knowledge, this is the first manuscript to evaluate different molecular responses (mRNA, miRNAs, global DNA methylation and hydroxymethylation, respectively) of bovine oocytes and embryos submitted to the LM culture system.

Initially we evaluated the impacts of the LM system during the IVM period. The IVM rate did not differ between groups, corroborating the finding by Ledda et al. [15] and Bebbere et al. [16] in two studies in sheep, where IVM rate was also similar between control and LM group. However, we observed a lower cumulus cells expansion in the LM group compared to the control, and this expansion is highly related to the oocyte’s ability to mature and fertilized [25, 26]. Next, we investigated transcript levels in cumulus cells collected from these oocytes, and observed a downregulation in three genes in the LM group compared to the control group: *EIF4E*, *BCL2* and *GAPDH*.

*EIF4E* is associated with cell differentiation and proliferation, due its function to mediate protein translation [27], thus the observed decrease in transcripts could be associated with a low response to IVM. *GAPDH* is a gene involved in cell metabolism, impacting cell energy production [28]. Recent studies demonstrated that addition of iodoacetate, a GAPDH inhibitor, during IVM could influence in embryo development, through a lower rate of cleavage and blastocyst, demonstrating the importance of the glucose metabolism mediated by *GAPDH* [29]. For this, the observed results in cumulus cells are possibly associated to a low cell metabolism during IVM. *BCL2* is a gene involved in blocking apoptosis in different cells type [30]. In women, *BCL2* mRNA expression was significantly higher in cumulus cells associated with mature oocytes than those associated with immature oocytes [31], suggesting that the reduced expression identified in our study could indicate that COCs did not have a proper *in vitro* maturation. Additionally, the investigated transcripts in the oocytes did not demonstrate to be affected by the LM culture system. Therefore, these gene expression results suggest that the IVM was negatively affected by the LM system, mainly due to decreased expression of important transcripts in cumulus cells.

As we observed a decrease in the expansion of cumulus cells in the LM group, it would be expected to also observe a reduction in the expression of *HAS2*, a gene highly associated with this process [32]. However, as gene expression is very dynamic to generate a phenotype, thus it is possible that the gene expression may already have been initiated, translated, and have its expression back to normal levels, while the phenotype still persists [33]. Perhaps, the action cascade that leads to an increase in *HAS2* gene expression may have already occurred at the time of sample collection, 22h after IVM. Corroborating with our work, Bebbere et al. [16], also investigated in the oocytes maturated in LM or conventional system the expression of the genes *BPM15* and *GDF9*, two genes that have critical role in oocyte maturation [34], and they also did not observed difference between the culture systems in oocytes from adult sheep.

We also investigated the effects of LM system applied during IVC. For that, presumptive zygotes were placed in the LM and conventional culture until day 7. The blastocyst rate was considerably reduced in the LM group. Another difficulty was to maintain the droplets intact until the end of the culture period. Due to that, the number of structures in LM system was smaller than in the conventional system. The same also occured with the drops during the IVM, however, as it was only 1 day in culture, we had a smaller loss of these microbioreactors, but during the 7 days culture this fragility in the system was more noticeable. The blastocyst rate obtained in control group corroborates with other studies [3, 21], demonstrating that the performance of the technique was efficient. In addition to the lower blastocyst rate, the blastocysts subjected to the LM system presented a lower number of cells, another indication of the effect of this system on early embryo development [35]. This was the first study to cultivate embryos inside microbioreactors produced by LM and we observed that the technique is feasible, since we obtained developed blastocysts, however it can limit embryonic development by reducing blastocyst rate.

In order to further understand the effects of the LM system during IVC, we investigated the bta-miR-615 and the global DNA methylation and hydroxymethylation. Our results demonstrated a decrease in the levels of miR-615 in blastocysts cultured in the LM system compared to the convetional system. The miR-615 has its transcript located in an intron region of the *Hoxc5* gene, this in turn is one of the best characterized genes belonging to a gene family called homeobox. In other words, genes that code for transcription factors [22]. These transcription factors regulate several other genes, especially at the embryogenesis phase, having a direct role in embryo development [36].

Interestingly, the global DNA methylation and hydroxymethylation was increased in embryos cultured in the LM system compared to the conventional system. The global DNA methylation and hydroxymethylation profile is influenced by the culture environment, as was demonstrated by Li et al. [37] and Bomfim et al. [38], where in both studies, global DNA methylation was increased in bovine embryos produced at high oxygen tension compared to low oxygen tension, demonstrating that culture environment can affect these epigenetics marks. Additionally, embryos developed *in vitro* show hypermethylation at several genomic loci compared to *in vivo* [39], which may partially corroborate with the differences we observed in the *in vitro* blastocyst development between our LM system. These results may indicate the modulation of embryo epigenetic marks according to the culture system, which can lead to an abnormal expression of certain genes, either repression or overexpression.

In summary, the LM system presents great challenges to be applied in the IVM and IVC. The IVM rate did not appear to be affected by the LM system; however, we observed changes in transcripts related to COCs function and lower expansion of the cumulus cells. Nevertheless, is important to highlight that the number of pools used in this manuscript are limited in order to avoid season variation, thus a large sample size could support even further our gene expression findings related to COCs quality. Additionally, we were able to produce blastocysts using the LM system even with the seven days culture challenge. However, the LM system decrease the number of blastocyst cells and miR-615 expression, while inducing an increase in global DNA methylation and hydroxymethylation. Thus, the LM system needs to be improved in order to be used in commercial laboratories of IVP.

## Conclusion

This was the first study in cattle that performed the IVM and IVC steps using the LM microbioreactors. Despite the difficulty we had in producing the LM droplets due to their fragility, which resulted in the loss of oocytes and embryos, we observed that the IVM rate was not altered. Importantly, the expression of transcripts associated with COCs quality and development were altered in cumulus cells. In embryo culture experiment, we had lower rate of blastocyst and lower number of blastocyst cells. This system was also able to decrease the expression of miR-615, requiring further studies to understand the role of this modulation in the development of blastocysts. Additionally, we had an increase in the patterns of global DNA methylation and hydroxymethylation in the LM group, which can modify the accesssability of genes to be transcribed. Thus, we consider these results promising since it was the first time that the LM system was used for embryo culture. Even with a lower number of blastocysts compared the control, the zygotes were able to develop until blastocysts in the LM system, which is very interesting given the great complexity to obtain blastocysts *in vitro* and the high fragility of the liquid marble structures. Additionally, the technique still needs multiple adjustments, such as temperature, media volume, media components, oxygen level, among others, to turn its use viable. Since all these factors have been adjusted for conventional 2D culture over the last 40 years, we believe that with the change of culture systems from 2D to 3D, these factors will also be adjusted to improve the blastocysts yields in the future.
